# Effects of *PTPN6* Gene Knockdown in SKM-1 Cells on Apoptosis, Erythroid Differentiation and Inflammations

**DOI:** 10.3390/cimb46110715

**Published:** 2024-10-28

**Authors:** Li Yu, Xiaoli Gu, Pengjie Chen, Rui Yang, Yonggang Xu, Xiupeng Yang

**Affiliations:** Department of Hematology, Xiyuan Hospital, China Academy of Chinese Medical Sciences, Beijing 100091, China; yuli0610@163.com (L.Y.); gxl102707@163.com (X.G.); c15515981402@163.com (P.C.); yangrui04190702@163.com (R.Y.)

**Keywords:** *PTPN6*, SKM-1 cells, myelodysplastic syndrome, apoptosis, erythroid differentiation

## Abstract

**Objective:** Protein tyrosine phosphatase non-receptor type 6 (*PTPN6)* is a cytoplasmic phosphatase that acts as a key regulatory protein in cell signaling to control inflammation and cell death. In order to investigate the role of *PTPN6* in hematologic tumor myelodysplastic syndrome (MDS), this study infected SKM-1 cell line (MDS cell line) with packaged H_*PTPN6*-shRNA lentivirus to obtain H_*PTPN6*-shRNA SKM-1 stable strain. The effect of *PTPN6* knockdown on apoptosis, erythroid differentiation, and inflammations in SKM-1 cell line was examined. **Methods:** The stable knockdown SKM-1 cell line was validated using qPCR and Western blot assays. The proliferation activity, apoptosi, erythroid differentiation, and inflammatory cytokines in SKM-1 cells were assessed before and after transfection. **Results:** qPCR confirmed that the expression level of H_*PTPN6*-shRNA in SKM-1 cells was significantly reduced, and Western blot showed that the protein expression level of H_*PTPN6*-shRNA in SKM-1 cells was also significantly reduced. The CCK-8 cell viability assay confirmed that stable gene knockdown did not affect cell viability. Flow cytometry revealed that the apoptosis rate of cells in the *PTPN6* knockdown group was 0.8%, lower than the 2.7% observed in the empty plasmid group; the expression rate of the erythroid differentiation marker CD235a was 13.2%, lower than the 25.0% observed in the empty plasmid group. The expression levels of the proinflammatory factors IL-6 and IL-8 increased, and the expression levels of the inhibitor factor IL-4 decreased. **Conclusions:** The *PTPN6* gene was successfully knocked down using lentivirus-mediated transduction, and the constructed cell line was validated using PCR and Western blot. The CCK-8 cell viability assay confirmed that stable gene knockdown did not affect cell proliferation viability. Flow cytometry analysis of apoptosis and erythroid differentiation indicated that *PTPN6* knockdown inhibits apoptosis and erythroid differentiation in SKM-1 cells and also alters the level of inflammations in the bone marrow microenvironment. It suggests that the *PTPN6* gene acts as a tumor suppressor in myelodysplastic syndrome cells, influencing hematopoietic cell apoptosis, erythroid differentiation, and inflammations. This provides a reliable experimental basis for further in-depth studies on the mechanism of *PTPN6* in MDS and related pharmacological research.

## 1. Introduction

Myelodysplastic Syndrome (MDS) is a typical malignant clonal disorder of hematopoietic stem/progenitor cells, characterized by abnormal development of bone marrow cells, ineffective hematopoiesis, peripheral blood cytopenia, and progression to acute myeloid leukemia [1]. Current conventional treatments for MDS include symptomatic supportive care, transfusion, immunosuppressive therapy, demethylating agents, and hematopoietic stem cell transplantation. Although a variety of treatment options can offer more refined and individualized strategies for MDS patients, existing MDS treatment approaches still face challenges such as drug resistance, bone marrow suppression, and high costs [2,3]. The 2016 WHO MDS diagnostic criteria introduced genetic mutations into the classification of MDS for the first time. Increasingly, research has shown that genetic mutations play a significant role in the onset, development, diagnosis, treatment, and prognosis of MDS, with 70–90% of MDS patients detected with at least one genetic mutation [4]. In our previous studies, we screened the *PTPN6* gene using methylation sequencing and transcriptome sequencing, combined with Gene Ontology (GO) and the Kyoto Encyclopedia of Genes and Genomes (KEGG) [5].

PTPN6 is a non-receptor protein tyrosine phosphatase containing an SH2 domain belonging to the PTPN family, specifically *SHP-1*. It plays an important role in regulating growth and proliferation [5]. Recent studies have shown that *PTPN6* is a key regulatory protein in cell signal transduction, controlling inflammation and cell death by inhibiting caspase-8 and *Ripk3/Mlkl*-dependent inflammation [6]. Additionally, *PTPN6*, through the *SHP-1/JAK2/STAT3* signaling pathway, plays an important role in the treatment of osteosarcoma, hepatocellular carcinoma, and leukemia [7,8,9]. Furthermore, *PTPN6* is considered a candidate tumor suppressor gene in hematologic malignancies and solid tumors, mainly expressed in the hematopoietic system and epithelial cells, and is involved in regulating hematopoietic cell signaling [10]. It has been widely reported that *PTPN6* is abnormally hypermethylated in MDS patients, leading to reduced expression [11]. The abnormal expression of *SHP-1* in MDS cells SKM-1 increases *STAT* protein phosphorylation, promoting cell proliferation and inhibiting apoptosis [12].

Given the significant role of *PTPN6* mutations in MDS, but with no existing studies showing a correlation between *PTPN6* knockdown in the MDS cell line SKM-1 and apoptosis, erythroid differentiation, and inflammations, we constructed a stable H_*PTPN6*-shRNA SKM-1 cell line by infecting SKM-1 cells with recombinant lentiviral plasmids and packaging plasmids. Our research found that *PTPN6* knockdown inhibits apoptosis and erythroid differentiation in the SKM-1 cell line and alters the levels of inflammatory factors in the bone marrow microenvironment, indicating that the *PTPN6* gene acts as a tumor suppressor in myelodysplastic syndrome (MDS) cells, affecting hematopoietic cell apoptosis, erythroid differentiation, and inflammations. This provides a theoretical basis for further exploration of the role of *PTPN6* in MDS and related target gene research.

## 2. Materials and Methods

### 2.1. Main Materials and Instruments

Fetal Bovine Serum (Cegrogen, Germany, lot: A0500-3010/P211102); DMEM(Viva Cell BIOSCIENCES, Rome, Italy, lot: C3110-0500/2243278); Penicillin G sodium salt (Meren, Suzhou, China, lot: MB2047/N1127A); Streptomycin sulfate (Sanjie, Shanghai, China, lot: 211001); MolPure^®^ Cell/Tissue Total RNA Kit Cell/Tissue Total RNA Kit (YEASEN, Shanghai, China, lot: 19221ES50); Hifair^®^ Ⅱ 1st Strand cDNA Synthesis SuperMix (YEASEN, Shanghai, China, lot:11120ES60); Hieff^®^ qPCR SYBR Green Master Mix (Low Rox Plus) (YEASEN, Shanghai, China, lot:11202ES08); Western HRP substrate (Immobilon, Shanghai, China, lot: A25742); Ammonium persulfate (APS) (Beyotime, Shanghai, China, lot: KR118); Skim milk Powder (Yili, Neimeng, China, lot: D8418); 12 cytokine reagent detection kits(RAISECARE, Shandong, China, lot: 20230602).

Fluorescence Quantitative PCR Instrument (9600Plus) (BIOER, FQD-96A, Shanghai, China); Ultraviolet-visible Spectrophotometer (NanoReady, FC-1100, Beijing, China); Metal Bath (Major Science, MK-20, Saratoga, CA, USA); Refrigerated Centrifuge (Thermo, ST40R, Vacaville, CA, USA); Western Blot Electrophoresis Apparatus (BIO-RAD, PowerPacm Basic Power supply #1645050, Hercules, CA, USA); Digital Display Dry Heater (Talboys, EQFO-949503, NJ, USA); Tanon 4600 Series Automatic Chemiluminescence/Fluorescence Image Analysis System (Tanon, 4600SF, Shanghai, China); Flow Cytometry (Beckman Coulter, NAVIOS, CA, USA).

The SKM-1 cells were kindly provided by Professor Chen Suning from the Department of Hematology, First Affiliated Hospital of Soochow University in Suzhou, China.

### 2.2. Cell Lines and Culture

SKM-1 cells were cultured in RPMI-1640 medium containing 10% FBS, supplemented with penicillin G sodium salt and streptomycin sulfate, growing in suspension. The 293T cells, which are used as packaging cells for lentivirus, are adherent epithelial-like cells cultured in DMEM containing 10% FBS. All cells were cultured at 37 °C with 5% CO_2_ and saturated humidity, and the seeding density was 5 × 10⁴ cells per ml in a 6 well, and 3 mL per well.

### 2.3. Construction of H_PTPN6 Interference Vector

Single-stranded DNA oligos containing the interference sequence were synthesized, annealed to form double-stranded DNA oligos, and then directly ligated into the enzyme-digested RNA interference vector using the pGMLV-SC5 RNAi vector. The ligation products were transferred into the prepared bacterial competent cells, and the growing monoclonal colonies were sequencing and identified for the successful target gene RNA interference vector.

### 2.4. Packaging of H_PTPN6 Lentivirus and Titer Determination

High-purity, endotoxin-free lentiviral vectors and their auxiliary packaging component vectors were extracted using the HG transgene reagent, and the constructed lentiviral vectors and auxiliary packaging component vectors were co-transfected into 293T cells. After 10–12 h of transfection, enhancing buffer was added, and 8 h later, the medium was replaced with fresh culture medium. After 48 h of continued culture, the supernatant containing lentiviral particles was collected, concentrated, and high-titer lentiviral concentrate was obtained. The number of fluorescent cells in each well was observed under a fluorescence microscope, and the virus titer was the number of cells expressing the fluorescence multiplied by the corresponding dilution.

### 2.5. Construction of Stable Cell Line by Lentiviral Infection

The packaged H_*PTPN6*-shRNA lentivirus was used to infect SKM-1 cells to obtain the stable H_*PTPN6*-shRNA SKM-1 cell line. On the first day, cells were cultured to the logarithmic growth phase, and 5 × 10⁴ cells were collected into a 1.5 mL EP tube, centrifuged at 800 r/min for 5 min, and the supernatant was discarded, leaving the cell pellet for later use. The required volume of lentivirus was calculated based on MOI = 300, and the virus was mixed with complete culture medium to a total volume of 300 μL, then added to the cell pellet, gently mixed without bubbles, and transferred to a 24-well plate. The plate was centrifuged at 2100 r/min for 30 min. After centrifugation, the cell culture plate was placed in a 37 °C, 5% CO_2_ incubator for overnight culture. On the second day, after 16 h of infection, cell status was observed. If black spots appeared on the bottom of the cell culture dish, the medium containing lentiviral particles was promptly removed and replaced with 500 μL of fresh complete medium; if the status was normal, the medium was supplemented to 500 μL. On the third day, the medium was replaced with fresh complete medium. On the fourth day, the cells were continuously cultured, observing for any abnormalities. On the fifth day, infection efficiency was assessed by observing fluorescence under an inverted fluorescence microscope, estimating the efficiency of lentiviral infection in target cells.

### 2.6. qPCR Evaluation of Target Gene Expression Changes

The expression levels of the target gene and reference gene in cell samples were detected by qPCR. The Ct values (threshold cycle number) of the target gene and the reference gene in each sample were obtained based on the qPCR reaction curve, and relative quantification was performed using the ΔΔCt method. Data analysis was performed using the LineGene9600 system from BIOER. Empty cells were used as control samples, and the expression of the target gene in each vector group was compared to calculate the expression effect.

### 2.7. Western Blot Detection of Gene Silencing Effect

Cells in the logarithmic growth phase were collected from the incubator, and the medium was removed. The cells were washed twice with pre-cooled PBS, then PBS was discarded. Cells were lysed on ice with RIPA lysis buffer, and 5× loading buffer was added. The protein samples were boiled in a water bath for 10 min, cooled on ice, and then directly loaded into the SDS-PAGE gel sample wells. Polyacrylamide gels were prepared for electrophoresis and membrane transfer and blocked using protein-free rapid blocking solution (Yarase, Shanghai, China, lot: PS108P). An appropriate amount of protein-free rapid blocking solution was added to the incubation box. The aker was shaken for 15 min, and then the primary antibody was incubated overnight (the dilution concentration of the primary antibody was 1:1000). The two antibodies were incubated the next day and developed after washing and the bands were retained.

### 2.8. CCK-8 Assay to Detect the Effect of PTPN6 Knockdown on Cell Line Proliferation Activity

The untransfected group, empty vector control group, and transfected group were seeded in a 96-well plate at a density of 1 × 10⁵ cells per well. At different time points (24, 48, 72 h), 10 μL of CCK-8 reagent was added to each well, and the absorbance (OD value) at 450 nm was measured using a microplate reader.

### 2.9. Flow Cytometry to Detect the Effect of PTPN6 Knockdown on SKM-1 Cell Apoptosis

Cells from each group were washed twice with PBS (2000× *g*, centrifuged for 5 min), and the cell pellet was collected. The pellet was resuspended in 500 μL of binding buffer, and 5 μL of Annexin V-FITC was added and incubated at room temperature in the dark for 10 min. Then, 5 μL of propidium iodide was added, mixed, and incubated in the dark at room temperature for 5 min. Samples were analyzed by flow cytometry to detect cell apoptosis.

### 2.10. Flow Cytometry to Detect the Effect of PTPN6 Knockdown on SKM-1 Cell Erythroid Differentiation

Each group of cells were washed twice with PBS (2000× *g*, centrifuged for 5 min) and the cell pellet was collected. The pellet was resuspended in 500 μL of binding buffer with 5 μL of CD235a and incubated at room temperature for 20 min. Erythroid differentiation was determined by flow cytometry after washing with PBS.

### 2.11. Flow Cytometry to Detect the Effect of PTPN6 Knockdown on SKM-1 Cell Inflammations

Cells in each group were centrifuged at 1500 r/min for 8 min, and 25 uL of serum was harvested and measured. Before the experiment, 12 cytokine reagents were set at room temperature, standards and samples were prepared according to the instructions, and serum levels of IL-5, IFN- α, IL-1 β, IL-4, IL- α, IL-12P70, TNF- α, IL-2, IL-6, IL-10, IFN- γ, and IL-17 were measured.

### 2.12. Statistical Analysis

Data results are expressed as mean plus or minus standard deviation (X ± SD), and statistical analysis was performed using GraphPad Prism9.0 and SPSS 26.0 software with *t*-tests or one-way ANOVA. Experiments were performed in at least three independent experiments for statistics. The comparison between the two groups was performed by t test, and the variance was homogeneous LSD test was used for inter-group comparison, and Dunnett’s T3 was used for inter-group comparison when variance was uneven. The rank sum test was used for skewness distribution. A *p*-value of <0.05 was considered statistically significant.

## 3. Results

### 3.1. PCR Expression of Target Gene PTPN6

**Figure 1:**
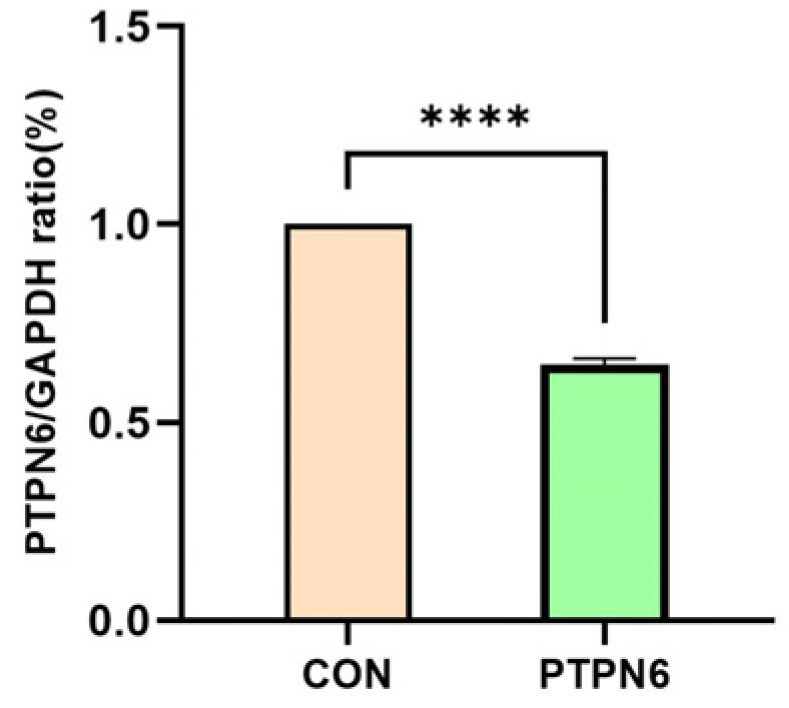
mRNA expression of target gene *PTPN6* in SKM-1 cell. (CON: Transfected with empty vector group; *PTPN6*: *PTPN6* transfected group; *n* = 3, X ± SD; Compared with the control group, **** *p* < 0.0001).

The expression levels of the target gene and the reference gene in the cell samples were detected by qPCR. The Ct values (threshold cycle number) of the target gene and the reference gene in each sample were obtained based on the qPCR reaction curve. The results are shown in Figure 1. SKM-1 cells were treated with transfected PTPN6. The result shows that compared with the control group, the mRNA expression was significantly decreased in the *PTPN6* group (*p* < 0.0001).

### 3.2. Construction Results of H_PTPN6 Interference Vector

**Figure 2:**
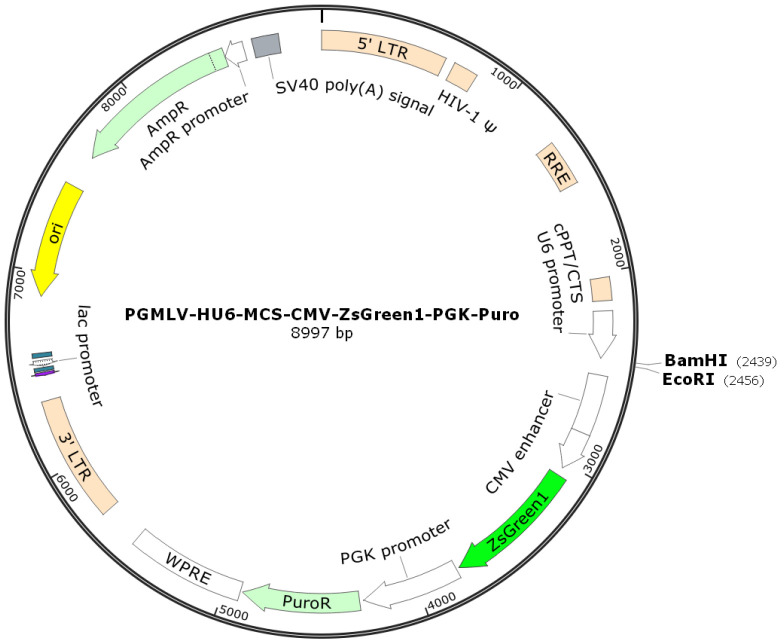
Carrier information of H_*PTPN6* Interference.

The vector was linearized by endonuclease restriction, and the target RNAi sequence was ligated into it to construct a vector carrying the target RNAi sequence. The vector information is pGMLV-SC5 RNAi, with the plasmid map shown in Figure 2. Based on the target gene sequence, multiple RNA interference target sequences were designed according to the RNA interference sequence design principles provided on public websites. Sequencing results of four kinds of shRNA interference vectors were shown in Table 1. The shRNA oligonucleotide sequences were then designed and synthesized according to the gene sequence. After comparison, the inserted fragment sequence in the recombinant clone was found to be completely consistent with the designed oligo sequence, confirming that the vector was successfully constructed.

### 3.3. Results of Packaging and Titer Determination of H_PTPN6 Lentivirus

**Figure 3:**
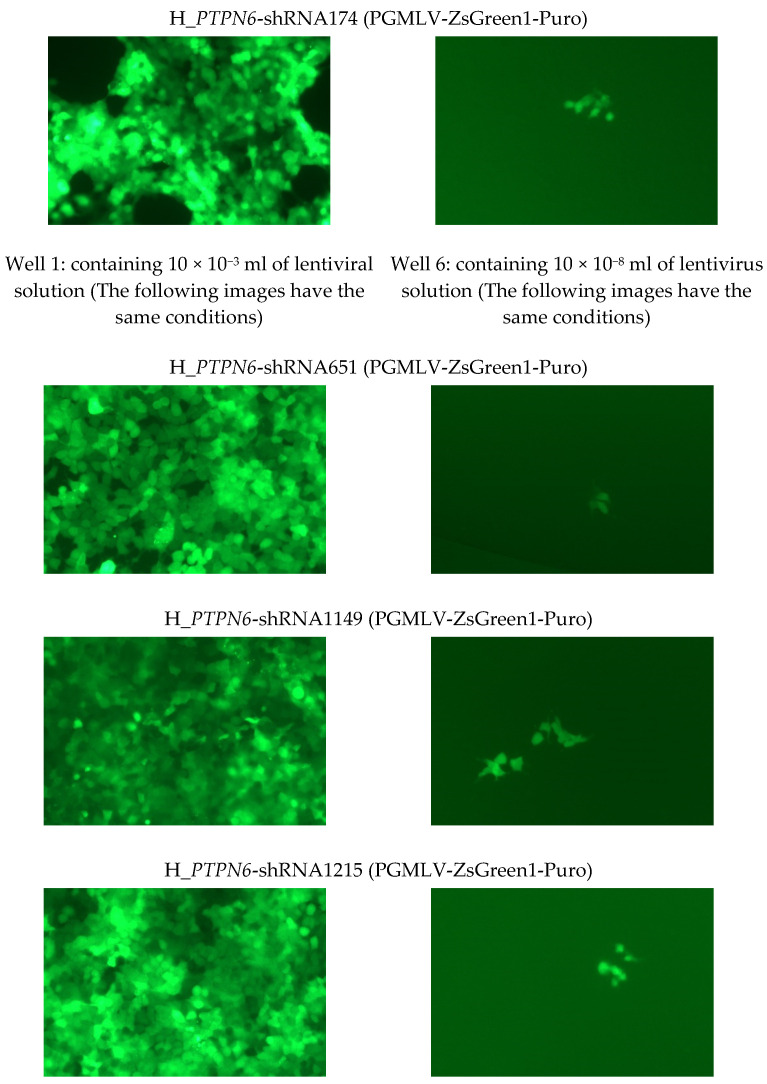
Fluorescence picture of partial wells (a field of view) 96 h of 4 H_PTPN6-shRNA after lentiviral infection (at least 50 cells expressing green fluorescence were observed in well 6, viral titer: 50TU/(10 × 10^−8^) mL = 5 × 10^8^ TU/mL).

The sequencing results of the four selected lentivirus clones and their titers are listed in Table 2. Fluorograms of the following portions of the wells (one field) were visible 96 h after lentiviral infection (Figure 3). The successfully constructed recombinant lentiviral plasmid and packaging plasmid were extracted using the plasmid extraction kit. The obtained plasmid DNA was dissolved in sterile TE, and its concentration and purity were determined by UV absorption, ensuring that the A260/A280 of the extracted plasmid DNA was between 1.8 and 2.0. The picture shows representative fields of view of four different clones/sh RNA after the infection with lentivirus. Over 50 cells expressing GFP were observed, showing successful Packaging of H_PTPN6 Lentivirus.

### 3.4. qPCR Evaluation of PTPN6 Knockdown Relative Expression Changes

**Figure 4:**
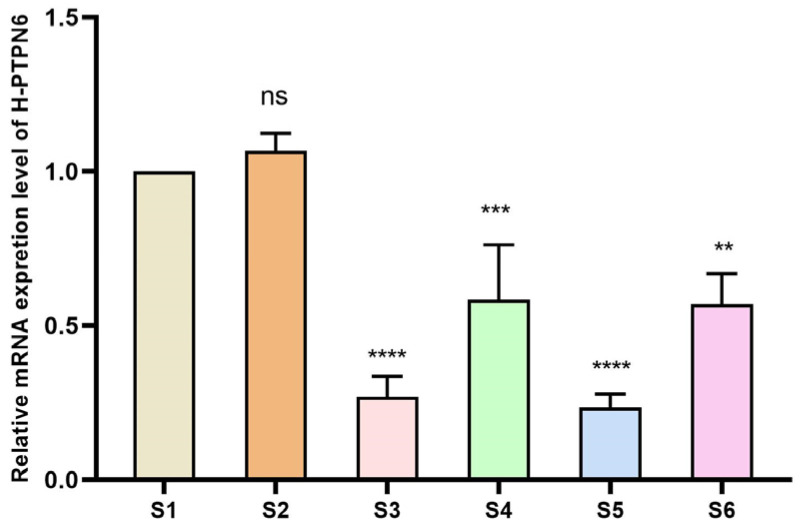
Relative expression of 4 H_*PTPN6*-shRNA after *transfection*. (S1: Control group; S2: Empty plasmid group; S3: H-*PTPN6*-shRNA174 group; S4: H-*PTPN6*-shRNA651 group; S5: H-*PTPN6*-shRNA1149 group; S6: H-*PTPN6*-shRNA1215 group. *n* = 3, X ± SD; Compared with the control group, ns: *p* > 0.05; ** *p ≤* 0.01; *** *p ≤* 0.001; **** *p ≤* 0.0001).

To verify whether the shRNA expression vector can continuously produce shRNAs in cells and thereby inhibit the relative expression of the target gene *PTPN6*, we performed qPCR on the samples. The differences were significant (*p* < 0.01), as shown in Figure 4. The relative expression of all four PTPN 6 transfected cells decreased, demonstrating successful transfection. Among these, the relative expression of H_*PTPN6*-shRNA1149 decreased most significantly, and the H_*PTPN6*-shRNA1149 SKM-1 cell will be used for subsequent CCK 8, apoptosis, and erythroid differentiation experiments.

### 3.5. Western Blot Results of PTPN6 Knockdown 

**Figure 5:**
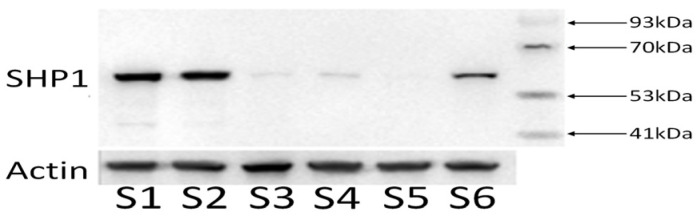
Expression of four proteins of the H_*PTPN6*-shRNA. (S1: Control group; S2: Empty plasmid group; S3: H-*PTPN6*-shRNA174 group; S4: H-*PTPN6*-shRNA651 group; S5: H-*PTPN6*-shRNA1149 group; S6: H-*PTPN6*-shRNA1215 group).

Cells in the logarithmic growth phase were collected and the protein expression in the knockdown cell lines was detected using Western blot. The molecular band size of *SHP 1* is 65 kD. Compared with the untransfected cell group (S1) and the empty plasmid group (S2), the protein expression in the transfected cell groups S3, S4, S5, and S6 was reduced (Figure 5), indicating that H_*PTPN6*-shRNA successfully transfected SKM-1 cells and effectively knocked down the *PTPN6* gene. Among these, H_*PTPN6*-shRNA1149 has the lowest protein expression, so this cell will be used for subsequent CCK 8, apoptosis, and erythroid differentiation experiments.

### 3.6. Effect of PTPN6 Knockdown on Proliferative Activity of Cell Lines

**Figure 6:**
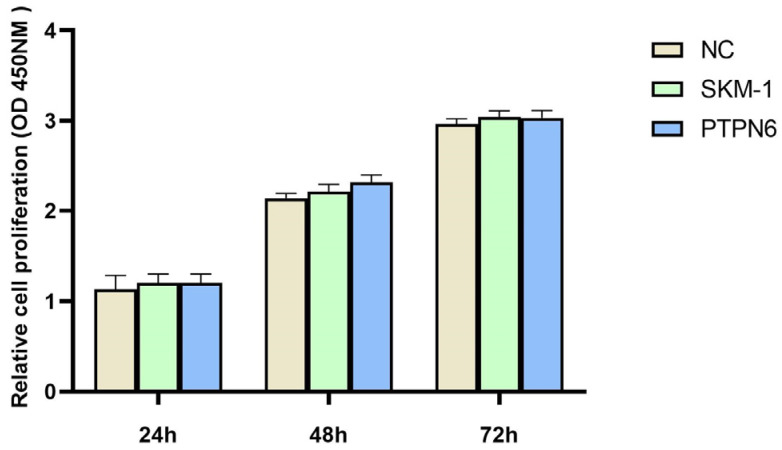
Cell proliferation activity was detected by CCK8. (NC: Empty plasmid group; SKM-1: SKM-1 cell group; PTPN6:H-*PTPN6*-shRNA1149 group. *n* = 3, X ± SD).

To verify whether the stable knockdown of *PTPN6* in cells affects cell proliferation viability, we measured the viability of untransfected, empty plasmid, and transfected groups at 24 h, 48 h, and 72 h using a 450 nm absorbance OD value for each well. As shown in Figure 6, the growth status of the cells in the three groups (empty plasmid, untransfected, and *PTPN6* knockdown) was generally consistent, and was statistically significant (*p* < 0.01). Thus, the stable knockdown of *PTPN6* does not affect cell viability and could be used for subsequent experiments

### 3.7. The Effect of PTPN6 Knockdown on Apoptosis and Erythroid Differentiation in SKM-1 Cells

**Figure 7:**
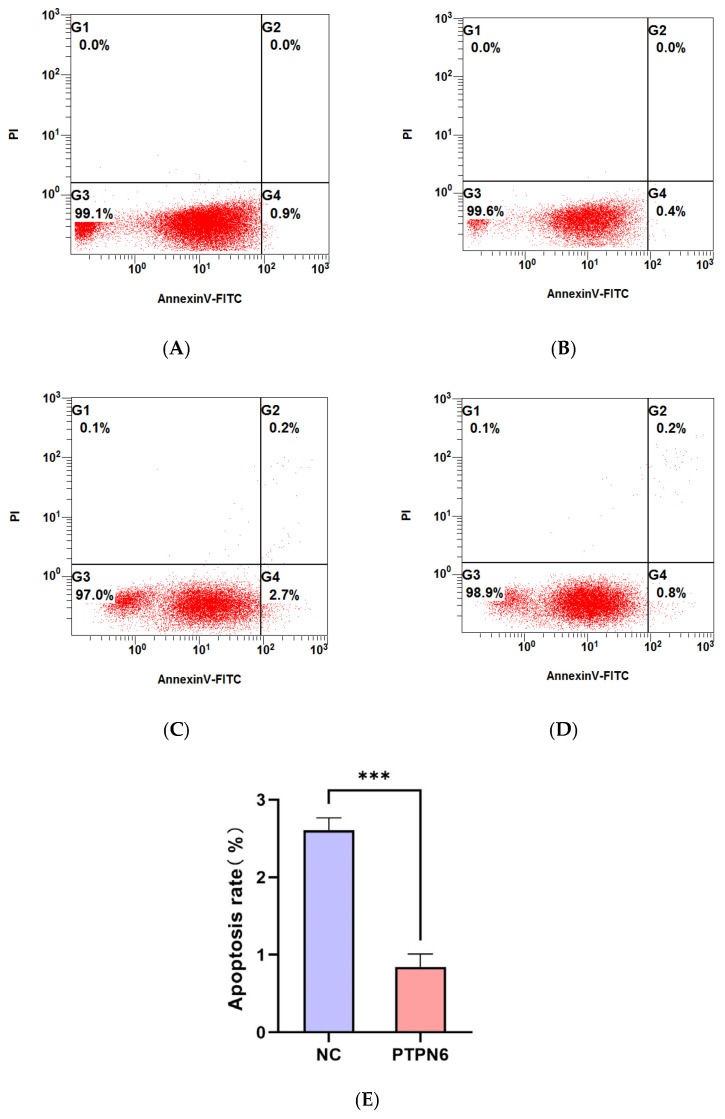
Effect of *PTPN6* knockdown on apoptosis in SKM-1 cells (NC: Empty plasmid cell; *PTPN6*: H_*PTPN6*-shRNA cell; *n* = 3, X ± SD, Compared with the empty plasmid group, *** *p ≤* 0.001). (**A**): Isotype control group of empty plasmid cell group; (**B**): Isotype control group of empty of H_*PTPN6*-shRNA cell group: (**C**): Empty plasmid cell group; (**D**): H_*PTPN6*-shRNA cell group; (**E**): Comparison of apoptosis rate between NC and PTPN6.

**Figure 8:**
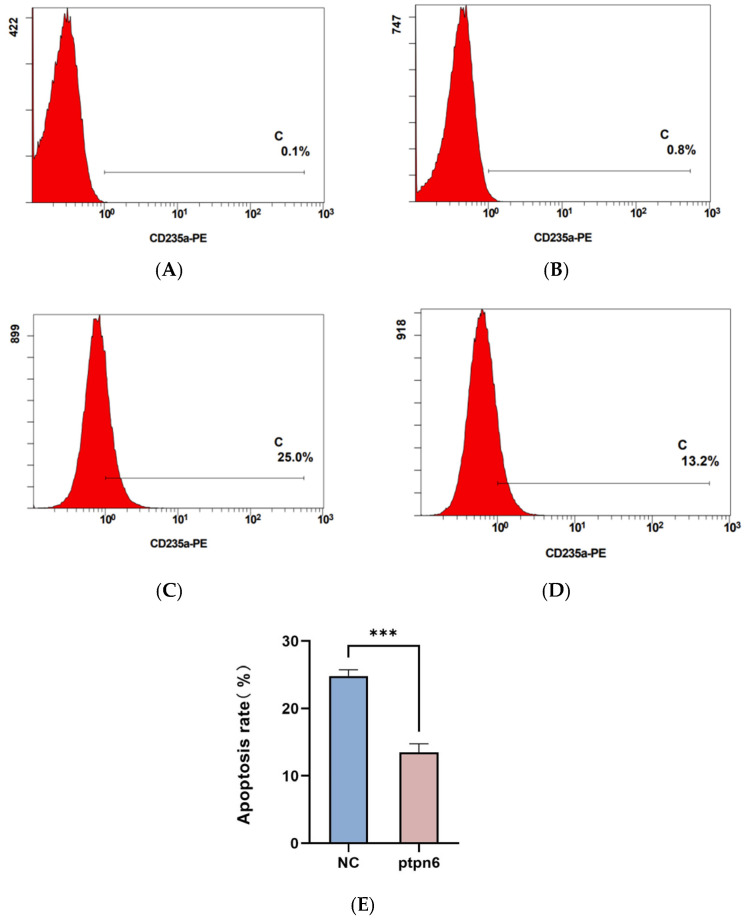
Effect of *PTPN6* knockdown erythroid differentiation in SKM-1 cells (NC: Empty plasmid cell; *PTPN6*: H_*PTPN6*-shRNA cell; *n* = 3, X ± SD, Compared with the empty plasmid group, *** *p ≤* 0.001). (**A**): Isotype control group of empty plasmid cell group; (**B**): Isotype control group of empty of H_*PTPN6*-shRNA cell group; (**C**): Empty plasmid cell group; (**D**): H_*PTPN6*-shRNA cell group; (**E**): Comparison of erythroid differentiation between NC and PTPN6.

Flow cytometry was used to detect apoptosis and erythroid differentiation in each group of cells, and each experiment was performed in triplicate. The rates of apoptosis and erythroid differentiation after PTPN 6 knockdown are shown in Figure 7 and Figure 8, respectively. The Isotype control group (Figure 7A and Figure 8A) and empty plasmid cell group (Figure 7B and Figure 8B) and H_PTPN6-shRNA cell group (cells without apoptosis reagents or any intervention) were used to determine the gating settings for flow cytometry. The apoptosis rate (Figure 7C) and erythroid differentiation marker CD235a expression rate (Figure 8C) in the empty plasmid group show that the apoptosis and erythroid differentiation expression rates are 2.7% and 25.0%, respectively. In the PTPN6 knockdown group, the apoptosis rate (Figure 7D) and CD235a expression rate (Figure 8D) show that the apoptosis and erythroid differentiation expression rates decreased to 0.8% and 13.2%, respectively, indicating that *PTPN6*, as a tumor suppressor gene, when mutated, increases the apoptosis rate and decreases the erythroid differentiation expression, leading to abnormal function of hematopoietic stem cells.

### 3.8. The Effect of PTPN6 Knockdown on Inflammations in SKM-1 Cells

**Figure 9:**
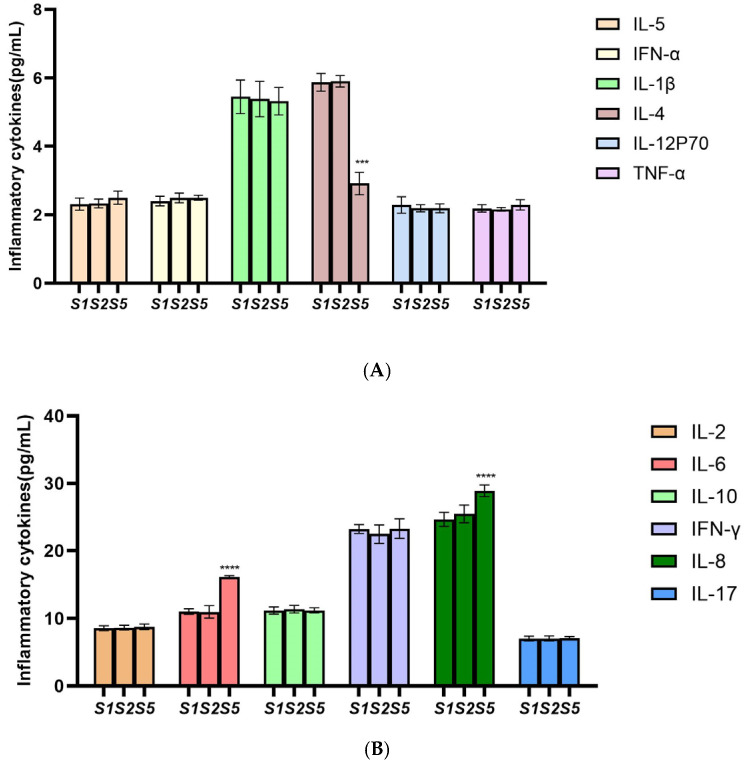
Effect of *PTPN6* knockdown erythroid differentiation in SKM-1 cells. (S1: Control group; S2: Empty plasmid group; S5: H-*PTPN6*-shRNA1149 group. *n* = 3, X ± SD; Compared with the control group, *** *p ≤* 0.001; **** *p ≤* 0.0001). (**A**)(B): Comparison of inflammatory cytokines in each group.

Flow cytometry was used to detect inflammations in each group of cells, and each experiment was performed in triplicate. The results of inflammations after *PTPN6* knockdown are shown in Figure 9. As shown in Figure 9A, the expression level of inflammation-inhibiting factor IL-4 was significantly decreased compared with control group, and as shown in Figure 9B, the expression level of pro-inflammatory factors IL-6 and IL-8 were increased. This suggests that *PTPN6* also plays an important role in regulating the inflammatory response and that its absence may lead to changes in the level of inflammation in the bone marrow microenvironment.

## 4. Discussion

The gene *PTPN6* (also known as *SHP-1)* belongs to the Protein Tyrosine Phosphatases (PTPs) family and is specific to phosphatidylinositol-3,4,5-trisphosphate (PIP3) [13]. *PTPN6* acts as a negative regulator of immune responses by hydrolyzing PIP3, and its deficiency leads to bone marrow proliferation and B-cell lymphoma in mice [14]. It is primarily expressed in hematopoietic cells, where it plays a negative regulatory role in cell signal transduction and is negatively correlated with tumor formation and growth [15]. Previous studies have found that promoter methylation of the *PTPN6* gene leads to its low expression in MDS patients, where it is considered a tumor suppressor gene. The *PTPN6* gene also regulates critical pathways such as *PI3K-Akt* and *JAK/STAT*, which are vital for many cellular functions [10,16]. It has been found that the *PTPN6* gene acts as a promoter in MDS and is highly correlated with methylation. Methylation of the *SHP-1* promoter significantly affects the prognosis of high-risk MDS patients, characterized by high methylation levels and low survival rates [17].

Apoptosis is a normal process of cell death that maintains the balance of cell numbers within tissues. It is achieved through programmed gene regulation, involving key regulatory molecules such as the Bcl-2 family proteins, the caspase family, and others [18]. *PTPN6*, through its phosphatase activity, can inhibit the signal transduction of multiple apoptotic pathways, thereby reducing the occurrence of apoptosis. For instance, *PTPN6* can silence apoptosis-activated cytokine receptors and related downstream molecules, inhibiting the activation of the apoptotic pathway [19,20,21]. It also negatively regulates B-cell apoptosis, playing a role in controlling the balance between B-cell survival and apoptosis [22]. Furthermore, the deficiency or dysfunction of *PTPN6* in immune cells may lead to excessive apoptosis, increasing the sensitivity of T cells, B cells, and macrophages to apoptosis, which could negatively impact immune responses [23,24]. *PTPN6* influences a range of molecules related to apoptosis regulation, such as members of the Bcl-2 family and caspase family proteins [6,25]. It is involved in the phosphorylation modifications of these key molecules, affecting apoptosis by regulating their expression and activity. Research has shown that the apoptosis rate of hematopoietic cells in the bone marrow of MDS patients is significantly increased. This apoptosis may be due to intrinsic genetic defects or external environmental factors leading to cell cycle regulation imbalance, causing premature death of hematopoietic cells [26].

One of the most common complications of myelodysplastic syndromes is anemia, which can be severe enough to lead to death. Despite the presence of sufficient or even excessive erythroid progenitor cells in the bone marrow, insufficient red blood cells are produced. Currently, ineffective erythropoiesis in MDS is believed to be due to differentiation arrest and increased apoptosis, making the promotion of terminal differentiation of erythroid progenitor cells key to resolving refractory anemia [27]. *PTPN6* plays an important regulatory role in the proliferation and differentiation of erythroid precursor cells, and its deficiency may lead to an imbalance in intracellular signal transduction, inhibiting normal differentiation of erythroid progenitor cells [28,29]. Additionally, its absence may affect the apoptotic mechanisms of erythroid precursor cells, leading to reduced cell survival and impacting red blood cell production [30].

*PTPN6* also plays an important role in regulating inflammatory responses, and its absence may lead to elevated inflammation levels in the bone marrow microenvironment, which may indirectly affect erythroid differentiation [31]. Cytokines play a key role in promoting cell-cell communication and interaction. Excessive production of inflammatory cytokines such as IFN- γ, TNF- α, IL-6, IL-IL-17, and IL-22 will promote [32,33]. Studies have found that inhibitory factors, such as [34,35,36] IL-10 and IL-4, are significantly increased in high-risk groups, that IL-2 levels are also different, and IL-2 and IL-4 levels will help predict MDS prognosis in patients. These inflammatory factor-mediated pathways lead to the growth of abnormal MDS stem and progenitor cells accompanied with inhibition of healthy hematopoietic [37].

Therefore, to determine the role of the *PTPN6* gene in MDS, we knocked down *PTPN6* expression in the SKM-1 cell line using lentiviral transduction, with pGMLV-SC5 RNAi as the vector and H_*PTPN6*-shRNA1149 selected to verify the effect of *PTPN6* knockdown. The results showed that the expression level and protein of *PTPN6* were significantly reduced after knockdown, and the gene sequence H_*PTPN6*-shRNA1149 was successfully constructed. CCK-8 assays confirmed that the viability and proliferation of the knockdown cell line were not affected by lentiviral transduction, ruling out interference for subsequent experiments. Flow cytometry analysis revealed that the apoptosis rate and erythroid differentiation expression rate in the *PTPN6* knockdown SKM-1 cell line were reduced, and the expression levels of the proinflammatory factors IL-6 and IL-8 increased, while the expression levels of the inhibitor factor IL-4 decreased, providing direction for our further exploration of MDS. The findings of this study contribute to a better understanding of the role of *PTPN6* in the tumor microenvironment and further demonstrate that *PTPN6* may be a potential target for slowing the progression of MDS.

This article excludes discussion on ethical approval due to its non-inclusion of human and animal usage.

## Figures and Tables

**Table 1:** Sequencing results of four selected shRNA vectors.

Sequencing Result	H_*PTPN6*-shRNA174(PGMLV-ZsGreen1-Puro)
GAAAGGACGAGGATCCGGATTTCTATGACCTGTATGGCTCGAGCCATACAGGTCATAGAAATCCTTTTTTAATTCTAGTTATTAATAGTAATCAATTACGGTTTCCACAAGATATATAAAGCCAAGA	
Figure Legend	shRNA Primers shRNA Target Sites
Sequencing Primer	Hu6-F: GAGGGCCTATTTCCCATGATT
Sequencing Result	0087_32723022500596_(71004GP-251)_[hU6-F]
Sequencing Result	H_*PTPN6*-shRNA651(PGMLV-ZsGreen1-Puro)
TTTCGATTTCTTGGCTTTATATATCTTGTGGAAAGGACGAGGATCCGGTGAATGCGGCTGACATTGACTCGAGTCAATGTCAGCCGCATTCACCTTTTTTAA	
Figure Legend	shRNA Primers shRNA Target Sites
Sequencing Primer	Hu6-F: GAGGGCCTATTTCCCATGATT CMV-R: GGGAACATACGTCATTATTG
Sequencing Result	71005GQ_hU6-F_TSS20230304-021-01952_A11 71005GQ_CMV-R_TSS20230304-021-01952_B11 (The final result is a splicing of the above two sequences)
Sequencing Result	H_*PTPN6*-shRNA1149(PGMLV-ZsGreen1-Puro)
AGAGGGACTCGTAGTATGTACGCGGACTCCATATATGGGCTATGAACTAATGACCCCGTAATTGATTACTATTAATAACTAGAATTAAAAAAGGAGCATGACACAACCGAATACTCGAGTATTCGGTTGTGTCATGCTCCGGATCCTCGTCCTTTCCACAAGATATATAAAGCCAAGA	
Figure Legend	shRNA Primers shRNA Target Sites
Sequencing Primer	CMV-R: GGGAACATACGTCATTATTG
Sequencing Result	0076_32723030100745_(71006GR-13) [CMV-R]
Sequencing Result	H_*PTPN6*-shRNA1215(PGMLV-ZsGreen1-Puro)
TTTCTTGGCTTTATATATCTTGTGGAAAGGACGAGGATCCGATTCGGGAGATCTGGCATTACTCGAGTAATGCCAGATCTCCCGAATCTTTTTTAATTCTATTTCCACAAGATATATAAAGCCAAGA	
Figure Legend	shRNA Primers shRNA Target Sites
Sequencing Primer	Hu6-F: GAGGGCCTATTTCCCATGATT CMV-R: GGGAACATACGTCATTATTG
Sequencing Result	0099_32723022500602_(71007GS-251)_[hU6-F] 0100_32723022500602_(71007GS-251)_[CMV-R] (The final result is a splicing of the above two sequences)

**Table 2:** The sequencing results of the four selected titers of *H_PTPN6* Lentivirus.

Lentivirus Name	Titer (TU/mL)
H_*PTPN6*-shRNA174 (PGMLV-ZsGreen1-Puro)	5 × 10^8^
H_*PTPN6*-shRNA651 (PGMLV-ZsGreen1-Puro)	5 × 10^8^
H_*PTPN6*-shRNA1149 (PGMLV-ZsGreen1-Puro)	5 × 10^8^
H_*PTPN6*-shRNA1215 (PGMLV-ZsGreen1-Puro)	5 × 10^8^

## Data Availability

The SKM-1 cell line was provided by Professor Chen and the PTPN6 expression vector was obtained from Genomeditech.

## References

[1] Hasserjian R.P., Germing U., Malcovati L. (2023). Diagnosis and classification of myelodysplastic syndromes. Blood.

[2] Volpe V.O., Garcia-Manero G., Komrokji R.S. (2022). SOHO State of the Art & Next Questions: Myelodysplastic Syndromes: A New Decade. Clin. Lymphoma Myeloma Leuk..

[3] Usuki K. (2022). [New treatment for myelodysplastic syndromes: Luspatercept and oral hypomethylating agents]. Rinsho Ketsueki.

[4] Arber D.A., Orazi A., Hasserjian R., Thiele J., Borowitz M.J., Le Beau M.M., Bloomfield C.D., Cazzola M., Vardiman J.W. (2016). The 2016 revision to the World Health Organization classification of myeloid neoplasms and acute leukemia. Blood.

[5] Sun S., Ma R., Hu X., Yang X., Xu Y., Wang H., Yang X. (2012). Karyotype and DNA-Methylation Responses in Myelodysplastic Syndromes following Treatment with Traditional Chinese Formula Containing Arsenic. Evid. Based Complement. Altern. Med..

[6] Tsui F.W., Martin A., Wang J., Tsui H.W. (2006). Investigations into the regulation and function of the SH2 domain-containing protein-tyrosine phosphatase, *SHP-1*. Immunol. Res..

[7] Speir M., Nowell C.J., Chen A.A., O’Donnell J.A., Shamie I.S., Lakin P.R., D’Cruz A.A., Braun R.O., Babon J.J., Lewis R.S. (2020). Ptpn6 inhibits caspase-8- and Ripk3/Mlkl-dependent inflammation. Nat. Immunol..

[8] Zhang T., Li S., Li J., Yin F., Hua Y., Wang Z., Wang H., Zuo D., Xu J., Cai Z. (2022). Pectolinarigenin acts as a potential anti-osteosarcoma agent via mediating SHP-1/JAK2/STAT3 signaling. Biomed. Pharmacother..

[9] Huang Y., Zhou B., Luo H., Mao J., Huang Y., Zhang K., Mei C., Yan Y., Jin H., Gao J. (2019). ZnAs@SiO2 nanoparticles as a potential anti-tumor drug for targeting stemness and epithelial-mesenchymal transition in hepatocellular carcinoma via SHP-1/JAK2/STAT3 signaling. Theranostics.

[10] Al-Rawashde F.A., Al-Sanabra O.M., Alqaraleh M., Jaradat A.Q., Al-Wajeeh A.S., Johan M.F., Wan Taib W.R., Ismail I., Al-Jamal H.A.N. (2023). Thymoquinone Enhances Apoptosis of K562 Chronic Myeloid Leukemia Cells through Hypomethylation of SHP-1 and Inhibition of JAK/STAT Signaling Pathway. Pharmaceuticals.

[11] Sharma Y., Ahmad A., Bashir S., Elahi A., Khan F. (2016). Implication of protein tyrosine phosphatase SHP-1 in cancer-related signaling pathways. Future Oncol..

[12] Han Y., Zhang J., Pang Y., Wang Y., Zhang X., Zhang H. (2021). The role of Src homology region 2 domain-containing phosphatase-1 hypermethylation in the classification of patients with myelodysplastic syndromes and its association with signal transducer and activator of transcription 3 phosphorylation in skm-1 cells. J. Int. Med. Res..

[13] Xu X., Yu Y., Zhang W., Ma W., He C., Qiu G., Wang X., Liu Q., Zhao M., Xie J. (2024). SHP-1 inhibition targets leukaemia stem cells to restore immunosurveillance and enhance chemosensitivity by metabolic reprogramming. Nat. Cell Biol..

[14] Mazgaeen L., Yorek M., Saini S., Vogel P., Meyerholz D.K., Kanneganti T.D., Gurung P. (2023). CD47 halts Ptpn6-deficient neutrophils from provoking lethal inflammation. Sci. Adv..

[15] Dempke W.C.M., Uciechowski P., Fenchel K., Chevassut T. (2018). Targeting SHP-1, 2 and SHIP Pathways: A Novel Strategy for Cancer Treatment?. Oncology.

[16] Zhang X., Yang L., Liu X., Nie Z., Wang X., Pan Y., Luo J. (2017). Research on the epigenetic regulation mechanism of the PTPN6 gene in advanced chronic myeloid leukaemia. Br. J. Haematol..

[17] Luo M., Xu X., Liu X., Shen W., Yang L., Zhu Z., Weng S., He J., Zuo H. (2022). The Non-Receptor Protein Tyrosine Phosphatase PTPN6 Mediates a Positive Regulatory Approach From the Interferon Regulatory Factor to the JAK/STAT Pathway in Litopenaeus vannamei. Front. Immunol..

[18] Zhang Y., Zhao D., Zhao H., Wu X., Zhao W., Wang Y., Xia B., Da W. (2012). Hypermethylation of SHP-1 promoter in patient with high-risk myelodysplastic syndrome and it predicts poor prognosis. Med. Oncol..

[19] Tsekoura G., Agathangelidis A., Kontandreopoulou C.N., Taliouraki A., Mporonikola G., Stavropoulou M., Diamantopoulos P.T., Viniou N.A., Aleporou V., Papassideri I. (2023). Deregulation of Autophagy and Apoptosis in Patients with Myelodysplastic Syndromes: Implications for Disease Development and Progression. Curr. Issues Mol. Biol..

[20] Baek S.H., Lee J.H., Ko J.H., Lee H., Nam D., Lee S.G., Yang W.M., Um J.Y., Lee J., Kim S.H. (2016). Ginkgetin Blocks Constitutive STAT3 Activation and Induces Apoptosis through Induction of SHP-1 and PTEN Tyrosine Phosphatases. Phytother. Res..

[21] Geraldes P., Hiraoka-Yamamoto J., Matsumoto M., Clermont A., Leitges M., Marette A., Aiello L.P., Kern T.S., King G.L. (2009). Activation of PKC-delta and SHP-1 by hyperglycemia causes vascular cell apoptosis and diabetic retinopathy. Nat. Med..

[22] Jung J.H., Yun M., Choo E.J., Kim S.H., Jeong M.S., Jung D.B., Lee H., Kim E.O., Kato N., Kim B. (2015). A derivative of epigallocatechin-3-gallate induces apoptosis via SHP-1-mediated suppression of BCR-ABL and STAT3 signalling in chronic myelogenous leukaemia. Br. J. Pharmacol..

[23] Chen J.L., Chu P.Y., Huang C.T., Huang T.T., Wang W.L., Lee Y.H., Chang Y.Y., Dai M.S., Shiau C.W., Liu C.Y. (2022). Interfering B cell receptor signaling via SHP-1/p-Lyn axis shows therapeutic potential in diffuse large B-cell lymphoma. Mol. Med..

[24] Ventura P.M.O., Gakovic M., Fischer B.A., Spinelli L., Rota G., Pathak S., Khameneh H.J., Zenobi A., Thomson S., Birchmeier W. (2022). Concomitant deletion of Ptpn6 and Ptpn11 in T cells fails to improve anticancer responses. EMBO Rep..

[25] Yam-Puc J.C., Zhang L., Maqueda-Alfaro R.A., Garcia-Ibanez L., Zhang Y., Davies J., Senis Y.A., Snaith M., Toellner K.M. (2021). Enhanced BCR signaling inflicts early plasmablast and germinal center B cell death. iScience.

[26] Kim B., Lee K.Y., Park B. (2017). Crocin Suppresses Constitutively Active STAT3 Through Induction of Protein Tyrosine Phosphatase SHP-1. J. Cell. Biochem..

[27] McBride A., Houtmann S., Wilde L., Vigil C., Eischen C.M., Kasner M., Palmisiano N. (2019). The Role of Inhibition of Apoptosis in Acute Leukemias and Myelodysplastic Syndrome. Front. Oncol..

[28] Platzbecker U., Della Porta M.G., Santini V., Zeidan A.M., Komrokji R.S., Shortt J., Valcarcel D., Jonasova A., Dimicoli-Salazar S., Tiong I.S. (2023). Efficacy and safety of luspatercept versus epoetin alfa in erythropoiesis-stimulating agent-naive, transfusion-dependent, lower-risk myelodysplastic syndromes (COMMANDS): Interim analysis of a phase 3, open-label, randomised controlled trial. Lancet.

[29] Forester C.M., Oses-Prieto J.A., Phillips N.J., Miglani S., Pang X., Byeon G.W., DeMarco R., Burlingame A., Barna M., Ruggero D. (2022). Regulation of eIF4E guides a unique translational program to control erythroid maturation. Sci. Adv..

[30] Cokic V.P., Bhattacharya B., Beleslin-Cokic B.B., Noguchi C.T., Puri R.K., Schechter A.N. (2012). JAK-STAT and AKT pathway-coupled genes in erythroid progenitor cells through ontogeny. J. Transl. Med..

[31] Cokic V.P., Bhattacharya B., Beleslin-Cokic B.B., Noguchi C.T., Puri R.K., Schechter A.N. (1999). SHP1 protein tyrosine phosphatase negatively modulates erythroid differentiation and suppression of apoptosis in J2E erythroleukemic cells. Biol. Chem..

[32] Bittorf T., Seiler J., Zhang Z., Jaster R., Brock J. (2022). Inflammatory Cytokine Profiles Do Not Differ Between Patients With Idiopathic Cytopenias of Undetermined Significance and Myelodysplastic Syndromes. Hemasphere.

[33] Nielsen A.B., Hansen J.W., Ørskov A.D., Dimopoulos K., Salem M., Grigorian M., Bruunsgaard H., Grønbæk K. (2022). Bone marrow-confined IL-6 signaling mediates the progression of myelodysplastic syndromes to acute myeloid leukemia. J. Clin. Investig..

[34] Liu Z., Xu X., Zheng L., Ding K., Yang C., Huang J., Fu R. (2023). The value of serum IL-4 to predict the survival of MDS patients. Eur. J. Med. Res..

[35] Kaniyattu S.M., Meenakshi A., Kumar M.B., Kumar K.R., Rao S., Shetty P.D., Shetty V., Shetty J.K., Shetty P.K. (2020). Cytogenetic and cytokine profile in elderly patients with cytopenia. Exp. Hematol..

[36] Kordasti S.Y., Afzali B., Lim Z., Ingram W., Hayden J., Barber L., Matthews K., Chelliah R., Guinn B., Lombardi G. (2009). IL-17-producing CD4+ T cells, pro-inflammatory cytokines and apoptosis are increased in low risk myelodysplastic syndrome. Br. J. Haematol..

[37] Gonzalez-Lugo J.D., Verma A. (2022). Targeting inflammation in lower-risk MDS. Hematol. Am. Soc. Hematol. Educ. Program.

